# The Value of Active Arts Engagement on Health and Well-Being of Older Adults: A Nation-Wide Participatory Study

**DOI:** 10.3390/ijerph18158222

**Published:** 2021-08-03

**Authors:** Barbara Groot, Lieke de Kock, Yosheng Liu, Christine Dedding, Janine Schrijver, Truus Teunissen, Margo van Hartingsveldt, Jan Menderink, Yvonne Lengams, Jolanda Lindenberg, Tineke Abma

**Affiliations:** 1 Leyden Academy, Rijnsburgerweg 10, 2333 AA Leiden, The Netherlands; kock@leydenacademy.nl (L.d.K.); jan.menderink@gmail.com (J.M.); ymmlengams@hotmail.com (Y.L.); lindenberg@leydenacademy.nl (J.L.); 2 Department of Medical Humanities, Amsterdam UMC, Location VUmc, De Boelelaan 1089a, 1081 HV Amsterdam, The Netherlands; y.liu3@amsterdamumc.nl (Y.L.); c.dedding@amsterdamumc.nl (C.D.); truusteunissen39@gmail.com (T.T.); 3 Sichting B.a.d., Talingstraat 5, 3082 MG Rotterdam, The Netherlands; janineschrijver@xs4all.nl; 4 Department of Occupational Therapy, Applied University of Amsterdam, Tafelbergweg 51, 1105 BD Amsterdam, The Netherlands; m.j.van.hartingsveldt@hva.nl

**Keywords:** long-term care, healthy and active aging, seniors, impact, elderly, arts activities

## Abstract

An emerging body of research indicates that active arts engagement can enhance older adults’ health and experienced well-being, but scientific evidence is still fragmented. There is a research gap in understanding arts engagement grounded in a multidimensional conceptualization of the value of health and well-being from older participants’ perspectives. This Dutch nation-wide study aimed to explore the broader value of arts engagement on older people’s perceived health and well-being in 18 participatory arts-based projects (dance, music, singing, theater, visual arts, video, and spoken word) for community-dwelling older adults and those living in long term care facilities. In this study, we followed a participatory design with narrative- and arts-based inquiry. We gathered micro-narratives from older people and their (in)formal caregivers (*n* = 470). The findings demonstrate that arts engagement, according to participants, resulted in (1) positive feelings, (2) personal and artistic growth, and (3) increased meaningful social interactions. This study concludes that art-based practices promote older people’s experienced well-being and increase the quality of life of older people. This study emphasizes the intrinsic value of arts engagement and has implications for research and evaluation of arts engagement.

## 1. Introduction

The corona disease 2019 (COVID-19) pandemic has shown the challenges of long-term care for older people worldwide [1,2,3,4]. It highlights the need for different solutions to improve the health and well-being of older adults and promote active aging. The field of arts and health movement aims to contribute to improving the health and well-being of, among others, older adults living at home, attending daycare, or living in a long-term care facility [5]. Both now, and before the pandemic, art projects have been launched in long-term care to improve the health and well-being of older adults in various countries [6,7,8,9].

An increasing number of studies, projects, and practices before the pandemic demonstrated the value of arts for health [10,11,12,13,14,15]. Many studies on the value of arts engagement regarding older people’s quality of life have focused on a particular outcome concerning well-being, ranging from clinical and psychological to social and spiritual outcomes [13]. While these studies indicate an emerging body of evidence, the evidence on arts for health and well-being is still fragmented and inconclusive. Policymakers, commissioners, and managers of health and care services often request robust and replicated evidence-based guidance on the role of the arts in improving health and well-being [16]. However, such robust guidance is not available, and the question is whether statistical evidence is appropriate in this field.

The scientific evidence is fragmented because most studies on the value of active arts engagement have focused on one specific activity, art-form, or group of participants (e.g., young people, people living in poverty, or people with dementia), and use either qualitative or quantitative methods [5]. Moreover, a shortcoming is that studies often focus on one domain of outcomes, either clinical, psychological, social, or spiritual. Furthermore, and maybe most importantly, they do not systematically consider the participants’ perspectives, implying that we still lack insight into the value of arts engagement from their perspective, mainly older people.

The purpose of this article is to contribute to the emerging body of evidence grounded in the experiences of arts engagement for older people. The article reports on a Dutch nationwide participatory study that combined narrative (qualitative and quantitative) and arts-based methods to map the value of arts engagement in 18 arts-based projects for older adults. The study aimed to explore the perceived value of active arts engagement. According to the participating older adults, we demonstrate that arts engagement produces (1) positive feelings, (2) personal and artistic growth, and (3) increased quality of social interactions. This study concludes that art-based practices promote older people’s well-being and increase the well-being of older people living in a long-term care facility, attending daycare, or living in the community.

## 2. Materials and Methods

### 2.1. Context of the Study

This study includes 18 art-engagement projects for older people (Table 1). In this case, the art projects, refer to activities organized by professional artists who operate outside of long-term care facilities or provide their services to such establishments. All projects in this study focus on active arts engagement (performing art activities, not just watching, or discussing artwork) with older adults in the Netherlands. All 18 projects were selected by arts and health funding bodies (RCOAK Foundation and Sluyterman van Loo Foundation) and a national health research and innovation organization (ZonMw) and received financial support for 18 months (January 2020 to July 2021) to continue their projects and contribute to the study. The selection criteria were the maximum variation in terms of the art form (e.g., dance, music, singing, theater, visual arts, video, and spoken word), geographical region (urban vs. rural, north, east, west, and south), variety of older adults (living independently, receiving (day)care, or living in long-term care facilities), and project scale (a small project has one facilitator with one group of participants in one location, a medium project has one or a maximum of five facilitators and groups of participants, usually all in a similar location and a large project has more facilitators, and activities and groups of participants all over the country).

### 2.2. Design and Methodology

To capture older people’s lived experiences of the value of active arts engagement, we followed a participatory design [17] with narrative- and arts-based inquiry [18]. This research approach is suggested to understand how individuals experience and interpret their everyday lived experiences [16,19,20]. In a participatory design, people who live and work on the topic of a study are involved in all aspects of the research [21]. In this study, a co-research group of seniors and a group of artists assisted the research team (see Supplemental I in Supplementary Materials). This engagement of the target group of our study is essential, as they bring an additional perspective, energy, and embodied knowledge to all phases of the research project [22,23,24]. The group of artists includes the same artists that led the 18 projects. They assisted the executive team members with access to the participants, activities, and interpretation sessions from their perspective and were involved in the arts-based inquiry. This last inquiry was initiated by the scholarly artist (J.S.) who is part of our executive research team.

### 2.3. Data Collection and Analysis

The data collection in the narrative- and arts-based inquiry consisted of five methods (Table 2), partly sequential and partly parallel. The data analysis was executed iteratively [25], which means that the analysis took place in different cycles during the data collection (Table 3). The interaction between the data collection and data analysis is summarized (Supplemental II in Supplementary Materials).

#### 2.3.1. Method Ia and Ib Including Inductive Analysis to Build a Conceptual Framework

The study starts with conducting face-to-face open interviews of 60–120 min with one or two artists of the art projects in this study (Method Ia). Interviews provide in-depth insight into contextual details and relevant information for this study. It provided insight into the artists’ perspectives about impact and mechanisms to achieve value. In these conversations, artists and researchers discussed ideas about necessary adjustments to our methods to suit the local context of initiatives. The aim was to use techniques that, for example, fit people with severe dementia and the different art forms. The interviews were audio-recorded and transcripted verbatim. The interviews were analyzed inductively using thematic analysis [25] using the data analysis software MAXQDA by a team of at least four researchers, who coded the data independently and discussed the findings with each other. The process of data gathering and analysis was an iterative process.

The first narratives from older adults were collected by formal interviews (Method Ib). This method is used to capture the lived experiences of older people concerning the value and meaning of arts engagement. Individual interviews offer an understanding into how individuals interpret their everyday lived experiences [25]. It provides an understanding of a previous experience that allows for an “insider view” and hence a deeper understanding of the issues of impact. In this interview, micro-narratives were gathered. A small list with open questions to share narratives was used for these interviews. The interviews were audio-recorded and transcripted verbatim. The interviews were analyzed inductively using thematic analysis [25] using MAXQDA. The aim of coding directly from the text data is to find new insights in the words and from the perspectives of older adults.

From the results of Method 1a and b, a conceptual framework was built (Supplemental III in Supplementary Materials), also using insights from the literature focusing on the value of arts and health [26,27,28,29,30,31] and the use of the concept of positive health [32] as a sensitizing concept [25] in constructing the framework. From this framework, a questionnaire for further quantitative framework analysis was built to identify patterns in the thick data considering the value of arts and health for older adults (Supplemental IV in Supplementary Materials). In choosing how to formulate the questionnaire and what language and definitions to use, it was essential to use words that emerged from these interviews as much as possible, so respondents would feel like the questionnaire represented their experience in their own words.

#### 2.3.2. Method II–V Including Deductive Analysis Based on a Conceptual Framework

From now all data were gathered in micro-narratives. Micro-narratives are brief expressions of respondents’ experiences (in text or audio, e.g., 10 to 15 sentences about their experiences, video, and photography). These were collected by various means: formal interviews (30–80 min) (Method II), 40 half-days of participatory observations with informal interviews (3–15 min) (Method III–IV), and online workshops with arts-based methods (Method V). This multimodality in narratives allowed for the expressive freedom and preferences of the participants involved, including those who were less language-oriented or cognitively able. From method Ia and Ib, a selection of narratives were included in the SenseMaker software.

To gather the narratives, researchers and senior co-researchers aimed to join activities of the art projects as participating members to gain a first-hand perspective of the activities. Rather than observing as outsiders, they acted as subjective observers. During and after the activity, they invited participants to share their experience ‘in the moment’ to gain an in-depth understanding of the embodied, practical, and physical experience. The informal and open-ended nature of the prompts allowed the person to share at their own pace comfortably. The researchers wrote detailed field notes during and after the event, and if relevant took photos and recorded videos (with the respondent’s consent). However, due to the restrictions and safety procedures of COVID-19, most narratives were gathered by telephone, sometimes after meeting each other in person (online or by a participatory observation on distance offline). These interviews were 30–80 min.

The questionnaire with signifier questions was used with SenseMaker, a data-software program that allows combining a multitude of qualitative data (answers to open questions and micro-narratives on personal experiences) with quantitative data based on the answers to the signifier questions [33,34,35]. Participants shared their micro-narrative and then answered about ten questions about their experiences. Participants interpreted their experiences in a structured way (self-signifying). SenseMaker discerns trends and patterns in an interacting system of data for complex settings. The triad questions are unique to SenseMaker. When reading the triads from the SenseMaker questionnaire, it is essential to consider that the dots representing the participants’ answers are stories that fit on a continuum between the three main themes identified in a question.

In addition, the scholarly artist organized three sessions on arts-based dissemination with artists (*n =* 16) to gain insights into the artistic, embodied and sensory experience of the art projects by participatory arts-based methods. The idea is that artists can help clarify what the art projects are aiming to do and how they may do this [18,19].

#### 2.3.3. Collaborative Analysis

The executive research team analyzed the micro-narratives with SenseMaker, created a presentation of the findings, and shared a micro-narrative book per art project with anonymous narratives with artists. This was followed by a collaborative analysis of the conclusions of 10 workshops with artists and project managers of the studied art projects (*n =* 39). The workshops aimed to facilitate a dialogue about the lessons learned on the value for older adults. Findings of this workshop were integrated with a second presentation of the results, which was discussed in an online validation session with various stakeholders (*n =* 56).

### 2.4. Participant Recruitment and Background Participant

The artists recruited participants for the formal interviews in the art projects. They used selection criteria: they were participants in one of the art projects in this study, and they could be interviewed by telephone. The main condition for eligibility for activities to be observed during participatory observations was that they were actively involved in participating in the art project at that moment. The artists helped link the researchers to participants during the participatory observations.

In total, 470 micro-narratives were collected. Older adults (n = 79) shared 419 micro-narratives in formal (Method II) and informal (Method IV) interviews. On average, one older adult shared five narratives per person. The older adults were 56 to more than 90 years old. In addition, 61% of the participants lived independently, 22% of the participants lived at home with some form of care, and 13% were residents of a long-term care facility. Although we did not ask the participants themselves, we estimate that 38% had dementia or other disabling condition. Furthermore, 78% of the older adults were female, reflecting the overrepresentation of women in art projects and among older adults in general. All were involved in one of the 18 arts engagement projects in this study. Older adults shared most micro-narratives, but 51 were shared by artists, care professionals, or family caregivers regarding the experience of older adults participating in projects (Method I and IV). A large group of older adults included in the observations had (severe) dementia and could not talk to the researchers because of COVID-19 safety rules.

## 3. Results

In this section, we present the highlights from the analysis of the experiences with the art projects: (1) arts participation brings positive feelings, (2) arts participation stimulates personal and artistic growth, and (3) arts participation creates meaningful social interactions. We present each theme with (parts of) micro-narratives, figures from the SenseMaker software, and finally, a selected photograph in which the experiences are captured in an arts-based way. Through the diverse ways of presenting the results (multimodal), we hope to share the nuances and values embedded in the broader cultural context. The results of phase A–E can be found in Supplemental V in Supplementary Materials. However, before we present the main themes from the analysis, we present some overall findings of this study, for example, the complexity of expressing value.

### 3.1. Complexity of Expressing Value

*“The intimacy that occurs, you can’t put that into words or examples. It is in the scenes that occur, it is also in the sharing of …. At one moment, somebody was going through a divorce after 40 years of marriage, and they wanted to share that with the group. And then you just see, that people dare to be vulnerable, if you dare to share that. So there is just a deeper layer which connects after a while. You cannot explain that to anybody.” (woman, 70–80 years, theatre)*

*“It gives me a good feeling inside, singing. I can’t really say anything about it besides that. For me it is hard to express this.” (woman, 70–80 years, singing)*

The above micro-narratives of participants indicate that they found it difficult to express the value of arts engagement in words. When a participant tried to capture the value of art in language, it seemed to dissolve and failed to cover the experience. The examples also illustrate that emotions, feelings, and interactions with people are interrelated and that art stretches beyond cognition and rationality, which is difficult to express verbally.

**Figure 1:**
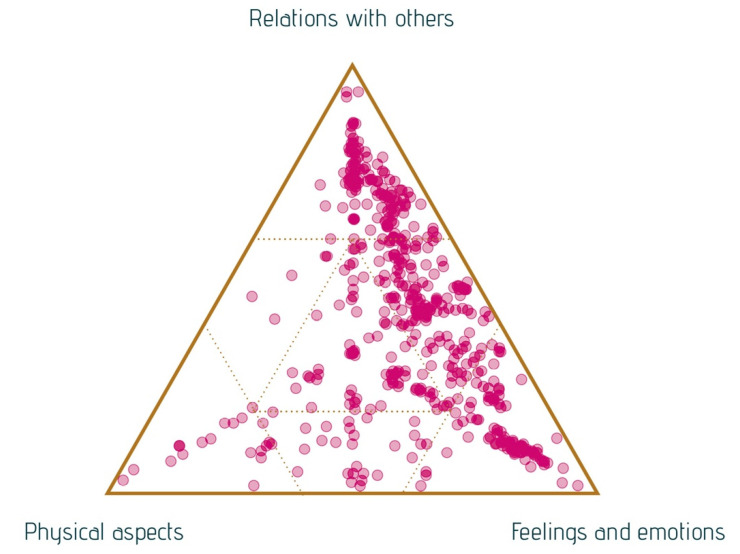
Main themes in the story triad (response to the question: What is most important in your experience?).

Even though it is difficult to capture value in words, we have tried to do so with narratives and analysis using SenseMaker. Listening to the stories, most of them referred to the value of arts engagement in terms of ‘feelings and emotions’ or ‘social relations with others.’ Some stories primarily discuss feelings, such as positive feelings, like happiness, zest for life, and relaxation. Other stories describe the intensity of social contact differently than normal. Only a minority of the micro-narratives focussed predominantly on physical elements of arts participation, especially the narratives about dancing. Figure 1 presents a pattern of dots of the micro-narratives in a triad. The dots are located close to the angles labeled ‘feelings and emotions’ and ‘relations with others.’ Nevertheless, some dots have been placed more in the middle, indicating that feelings and social and physical aspects play a part in the story.

### 3.2. Positive Feelings

A feeling of being alive and connected with their inner being was central in many stories. As noted, most participants found it hard to recall what it was like being in the arts activity and found it challenging to put their experience of that moment into words. For them, it was an experience that touched a deeper layer of consciousness beyond cognition and rationality.

*“...You are amongst people, you get to know each other a little bit and one person does it this way, the other that way and then we always have to laugh…. She (dance teacher) acts truly crazy, but really fun. Then she’ll laugh and say: Just pretend you have a snowball in your hand. Now throw it away quickly. And hit someone with it”, that is what she does. Then we all laugh. And we put some extra power into it, you enjoy it then, it is intense.” (woman, 80+ years, dance)*

**Figure 2:**
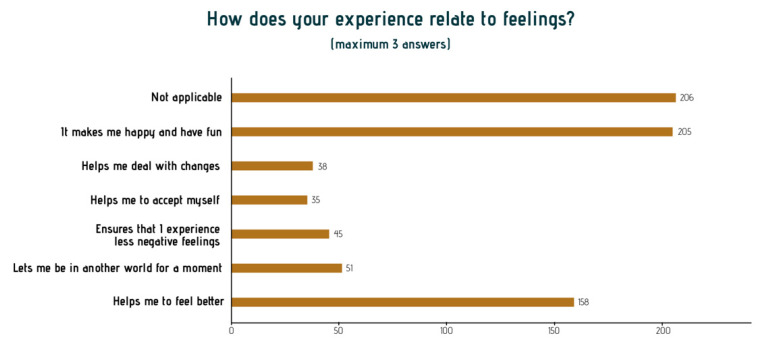
Main themes in the stories about ‘feelings and emotions’.

**Figure 3:**
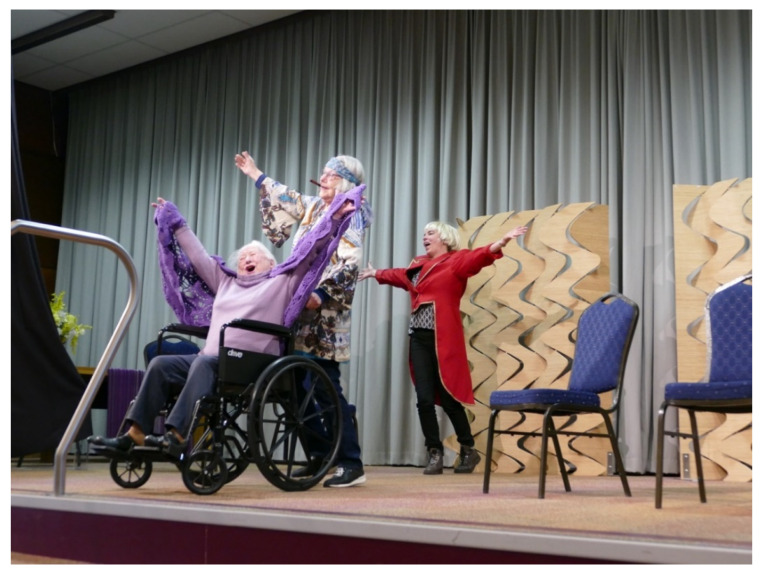
Participants living in the community playing theater for residents in a long-term care facility (photographer Henny Kallisvaart).

Most narratives are signified as ‘let us be happy and have fun’. Further analysis of this positive sensation of arts engagement demonstrated that participants describe a sense of being fully in the moment. The playfulness of the interactions with other participants and the artist makes people laugh and was described as feeling like they were young(er), even as young as if they were a child again. Because people were caught up, in the moment of making art together, it made them forget their limitations, worries, or physical ailments. They were able to let go of some of these issues and enjoy the moment, which most participants identified as being a precious experience at their age. They are described as ‘feeling better.’ Figure 2 and Figure 3 illustrate these feelings.

### 3.3. Personal and Artistic Growth

Being challenged is a second core aspect of the micro-narratives. Respondents liked being challenged by the artist or specific material and developing new skills, such as dance moves, performance, or artwork. They are engaged in the activity, focusing on learning artistic skills.

*“I really have progressed with what I am doing. And also, I am becoming looser in what I do. Or it is getting easier, you know, it flows more. In the beginning, you are very much occupied with: what am I making now, and what does it look like?” (woman, 70–80 years, visual arts)*

*“So then I ended up there. In the beginning I was really nervous: oh, can I really do that? A little bit fearful. I am scared I’ll spoil it or something. My hands started shaking. But slowly, the more mistakes you make, the more you learn. I was scared. That it would go wrong. That it would not become beautiful. That it will fail. My hands were shaking, like woooohhh!” (woman, aged 80+ years, visual arts)*

Many participants face challenges when starting with participatory arts, such as stepping out of their comfort zone or being challenged physically.

*“The singing is a challenge for me especially, because since I have been diagnosed with Parkinson’s disease, I could not sing anymore. No sound came from my mouth anymore. So then, we have a couple of songs, last time we had to sing, but I could not produce sound. I was quite taken aback that I could not do that anymore. I thought ‘Has Parkinson taken that away too?’ So I set myself to learning how to sing again. I spoke to my speech and language therapist and in bed at night I would move my head forwards and back and I felt that there was air coming in and I could sing again, that made me so happy! And then I practiced every day… I have taught myself how to sing again actually. So that is fun, when that works out.” (woman, 60–70 years, video)*

**Figure 4:**
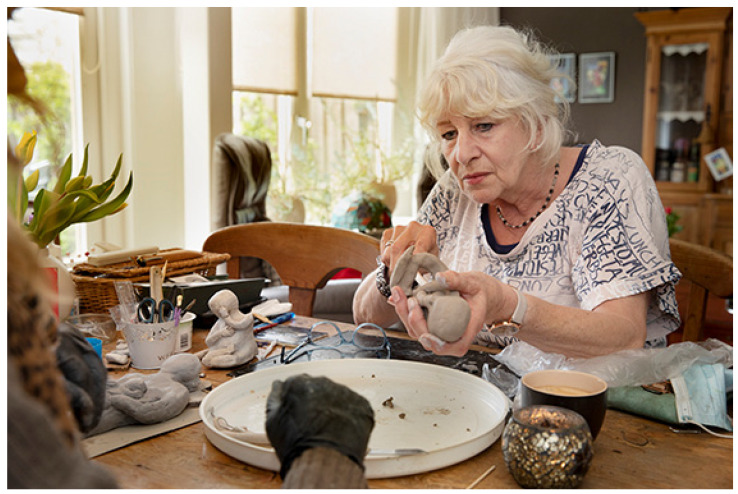
A participant doing visual arts with an artist at home at her kitchen table (photographer Janine Schrijver).

Most participants discover that they are capable of making art, sometimes with the help of the artists or others, even with an ‘aging body or mind’ (Figure 4). Participants refer to broader socio-cultural discourses that inform older adults on their bodies, minds, and aging. They go against what ought to be ‘safe’ for aging bodies. Trying out and doing new activities might provoke emotional reactions of fear, and once successful, feelings of self-confidence.

**Figure 5:**
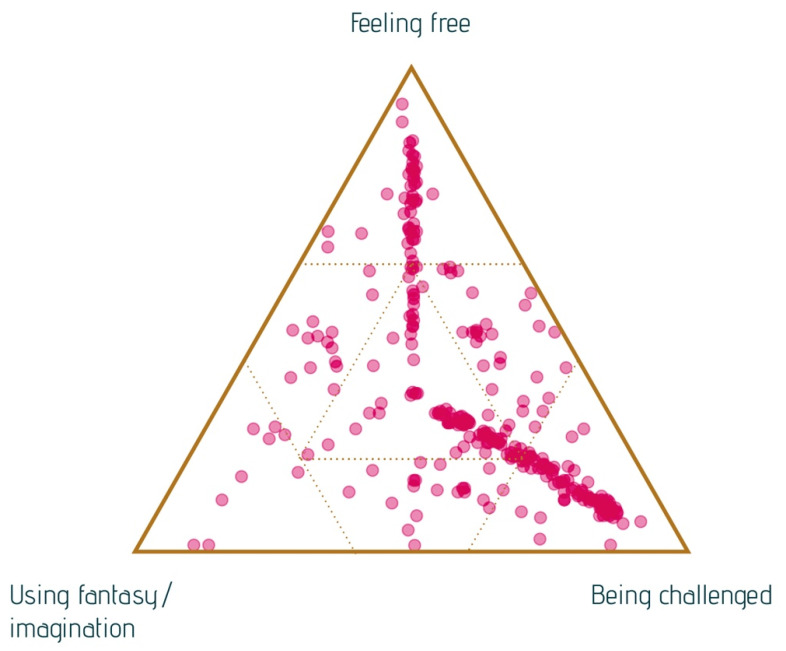
Triad on creative aspects of art engagement (response to question: Which aspects of creativity do you see in your experience?).

The feeling of being challenged (Figure 5) is often linked to the sense of meaning they felt in their lives. For example, participants of a theatre company rehearsed for a play they performed for people living in long-term care facilities (Figure 3), which gave them a purpose and made them feel fulfilled and valuable to other people. Participants explained that the art project gave them a reason, a concrete aim to practice their skills at home, improving their singing, for instance, of a song they were expected to perform in a film. In summary, people grow not only artistically, but also in a personal way, such as having an attitude of letting go and increasing self-esteem.

### 3.4. Meaningful Social Interactions

As a final theme, the results of this study indicate that arts engagement brings meaningful social interactions with others beyond the mundane, everyday interaction. Participants qualify these interactions as being of a different nature than other interactions. It could concern interaction with the artist, the other participants in the group, or sometimes the audience of a performance or exhibition such as loved ones or care professionals. The micro-narratives indicate that participants appreciate the social interactions arts activities offer them.

*“In other groups, we don’t get closer together, you don’t feel like it is a group. While here, even though I don’t know everyone by name, you still feel like you have taken part in a group experience when you go home…The atmosphere is important, the people are more important than the assignments…That mainly has to do with the challenge, see each other. Look each other into the eyes, so you encounter each other’s soul as it were. I don’t often look other people straight in the eye, or they look away, here you do really look at each other’s eyes.” (woman, 70–80 years, dance)*

**Figure 6:**
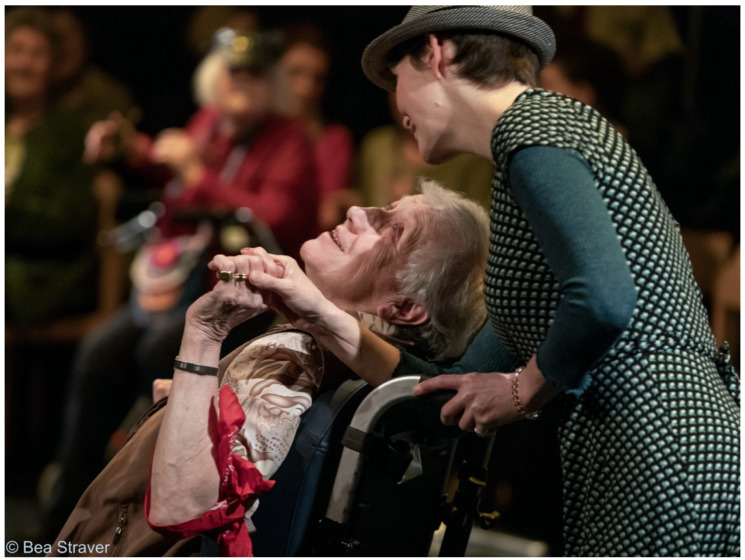
A participant dancing together in a long-term care facility (photographer: Bea Straver©).

In some cases, this interaction is physical, for example, in dance or theater activities, but it may also entail a shared moment of joy and mutual understanding. Moreover, participants indicate a sense of feeling connected to other participants. They share experiences of significant life events and jointly give meaning to these experiences through art. Making meaning provides a connection on an emotional level. They describe it as sharing their lifeworld with others without necessarily needing words. They produce the connection through bodily sensations and affects. Participants appreciate these personal interactions, and it creates a sense of belonging (see Figure 6). They truly see one another and even connect to each other’s souls. In comparison, they state that regular interactions in daily life can feel more superficial and unsatisfactory.

*“Singing together can be beautiful… there is a certain contact which you do not have in your daily life, this you find there. Feeling connected…” (woman, 70–80 years, singing)*

**Figure 7:**
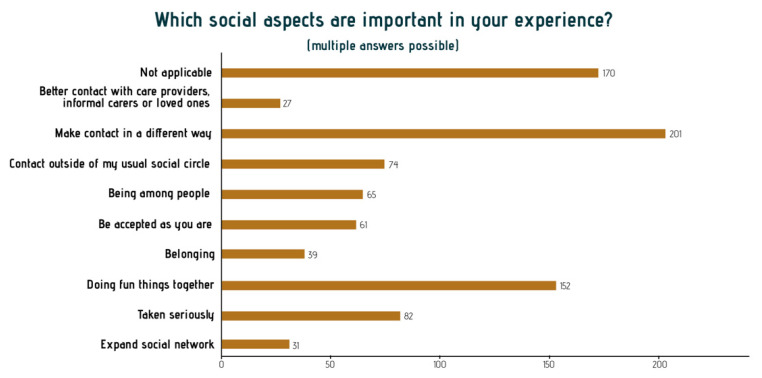
Main themes in the stories about social connections.

These interactions allow a different kind of connection: ‘Establishing contact with others in a different from usual way ‘through’ ‘doing things together,’ as illustrated in Figure 7.

## 4. Discussion

**Figure 8:**
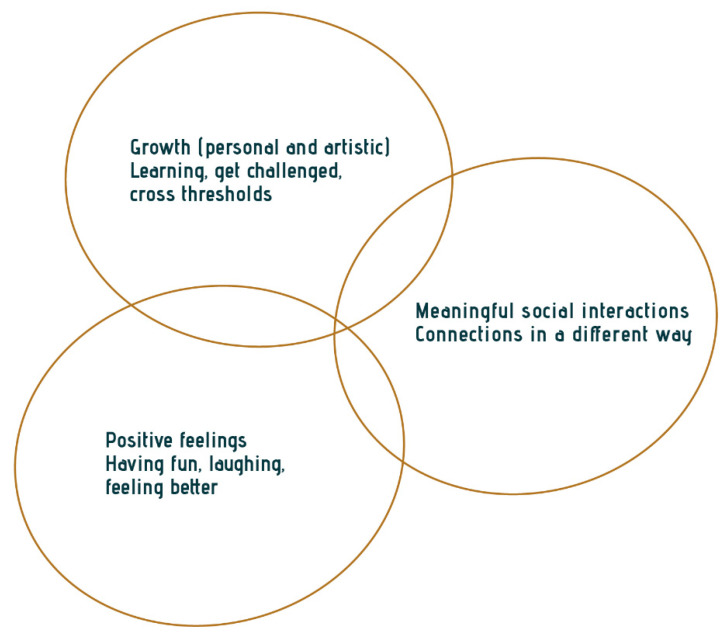
Three core aspects of the experienced value of active arts-engagement according to older adults.

This study provides insights into the value of active arts engagement from older participants’ perspectives, which focus on (1) positive feelings, (2) personal and artistic growth, and (3) more meaningful social interactions (Figure 8). These findings corroborate the literature on active arts engagement for older people and indicate the complexity of expressing the value for older adults themselves. In the literature, most impact frameworks are written in academic or medical language. This study presents three core aspects of the experienced value grounded in the perspective of older adults and described in their language. This language differs from the expert-driven and sometimes overly detailed frameworks or overviews [27,29,30] written in medical jargon [13,27,29,30] or organizational policy language [30].

Our study adds to the existing frameworks because it emphasizes the intrinsic value of active arts engagement. Some existing frameworks focus on the influence of arts engagement in terms of the values that lie outside art [13,29,30]. These frameworks employ means-ends thinking where the arts are considered a means to realize a specific external end or goal. In other words, they view art as an instrumental value. In our study, the participants stress that arts engagement is meaningful for older people, showing that art has intrinsic value. Being engaged in art and experiencing the flow in the moment is in and of itself meaningful for older people. This non-instrumental approach sheds new light on arts engagement and has serious implications for the research and evaluation of arts engagement. It resonates with the philosophy of art as described by Hans-Georg Gadamer. He pointed out that art has a ‘goal-less rationality’ that is essential for our human existence; its rationality lies in making art, not in external goals [36].

First, active art engagement increases older people’s emotional state [13]. Having fun and laughing have a direct effect on the body and state of mind. It relates the experience of a positive affect to a sense of liveliness and vitality: being wholly absorbed in a pleasurable moment makes one feel truly alive [35]. In the moment of experiencing this sensation, people are more open to contact with themselves, others, or ideas. For older adults, this sense of connection can be considered a feeling one still has a place in the world and still matters. Thus, we see that ‘art brings pleasure’ does not mean that it is ‘something for on the side’ or ‘for fun’ but can make an essential contribution to a meaningful life. Affect makes one feel connected to oneself and the surrounding world and provides a sense of place in this world, allowing one to take up space in it [36]. Enjoying life and experiencing happiness are central aspects of the quality of life [37]. The importance of this aspect seems somewhat underexposed in the literature.

Second, this study reveals that older adults are challenged and learn new skills when participating in art projects. It is a journey without a map: a semi-structured journey where they may experience unease. Engagement with an art form is described as a form of play that brings some tension, joy, confidence, and embodiment [35]. The metaphoric comparison of art with play comes from philosopher Gadamer [36]. He stressed that, in art, like play, there is always tension related to the material one is working with and to the chance of losing control of the play. This tension brings about seriousness and discipline not steered by external goals. In other words, the art as play is a goal in itself (=intrinsic value) and challenges participants to develop themselves to keep control of the game. By accepting the challenge, older adults claim to experience restoration from oppressive preconceptions about how they should behave as older adults. By experiencing affects of joy, older people can break free for a moment from set ideas of how they should behave or how society views them. Because artists offer older people the chance to experience affect and convey affect through their produced artifacts, art projects can possibly contribute to a more positive image of older adults and their role in society [38] (p. 125), changing dominating narratives on aging, long-life learning, and active aging.

Another important finding was that older adults appreciate the quality of social interactions that active art engagement provides. These meaningful interactions are often scarce in everyday contact with people, according to the participants. These daily interactions are superficial and do not resonate on an emotional and existential level, as the arts can. Interestingly, in existing literature, social outcomes of active art participation often focus on ‘expanding the social network’ [28] or ‘reduced loneliness and isolation, enhanced social support’ [12]. However, in our analyses, older adults focus on the emotional intensity of the moment of contact around existential matters, such as feelings of loss and regret, rather than the frequency of seeing people or the number of people in their social networks. Even though they do not know the names of those who share these experiences, they feel deeply connected. They significantly influence the quality of the moment of connecting, which gives them a sense of truly being seen and seeing the other person on an existential level [39].

Artists work with a certain intensity and openness to what cannot be predicted and controlled beforehand. They pay attention to the person, situation, and artistic and creative process. They create the possibility to experience affect and to be touched. Older adults describe this as being able to truly be themselves by fully expressing themselves in the art form of their choice.

Older adults often experience a kind of momentum, an atmosphere where they feel safe, accepted, and free. It is described as a place where people speak the same language, mutuality, and resonance. This usually does not concern a ‘linguistic’ or traditional language of art, but the aesthetic language (i.e., the sounds, image, movement, and scene). Everybody is unique because people experience affect in their own ways [40]. When we examine this value from the perspective of older people (e.g., people who develop problems in their cognitive functioning, such as people with dementia), it becomes even more apparent that affect can be valuable in maintaining the quality of life. Because it is a ‘language’ that does not necessarily require ‘normal’ cognitive functioning, but it can be experienced through the body, the senses, the subconscious [38]. In this way, affect forms a way of staying in touch with this group of people and expressing themselves to the world and people around them.

Finally, it is interesting that bodily functions are barely found in this study or described in the narratives of older adults. Most studies in which health and art come together have indicated positive results, particularly on these aspects, such as reducing pain [13,29]. While the participants did not refer to improvements in their objective health and body, nor signify the physical aspects as important in their experiences, their descriptions demonstrated that they did appreciate the value of arts activities, especially dance, music, and theater, in their subjective bodily experience, such as feeling more fit and energized. The results point to a distinction made in the philosophy of the body by Merleau-Ponty [41], who distinguished between the objective body (Körper) and subjective body (Leib). The discussions in this study with artists about this topic suggest that engaging in art might have a more significant value concerning the subjective experience of the body than on the objective body. However, the volume of micro-narratives concerning this aspect is small in this study. Further research into the value of arts on the embodied experience is therefore recommended.

This study had several limitations. First, gathering the narratives of older adults with cognitive disabilities was difficult, especially during COVID-19 pandemic. In the severe stages of dementia, people are less verbal and less coherent in speech, which demands creativity, such as working with images, and close observation of non-verbal communication. We sometimes needed to work with a proxy in the form of a health professional or someone who provided informal care. Second, we worked with diverse art projects (different art forms, various older adults, a diversity of local contexts). The question is whether we can do justice to this plurality and aggregate the findings at a more general level regarding the value and mechanisms of influence. Third, a critical point might be that charitable organizations selected the art projects. Although the selection criteria seem relevant, the research team was not involved in the final selection and might have made different choices. For example, not all projects involved individuals with mental health issues and people with a migrant background or a lower socio-economic status. Lessons about the value for these participants could have been of great interest (amongst others [42]).

This study reveals the challenge of describing the value of active arts engagement for older adults from their perspectives. Evaluating arts ‘interventions’ using external goals runs the risk of not capturing the essence of art as experienced by those participating in the arts. This essence can hardly be captured in words or figures as it emerges in the moment. We recommend further arts-based participatory research approaches to illuminate the value of arts engagement for older people.

## 5. Conclusions

This study used an innovative methodology to conduct research together with those who are involved in the topic of the study. This is a participatory research study together with older adults and artists. It emphasizes the intrinsic value of active arts engagement. This non-instrumental approach sheds new light on arts engagement. Overall, active arts engagement affects older adults with an intense sensation of being wholly absorbed in joyful moments, being challenged personally and artistically, and forming deep connections with the people sharing these moments. Affect makes older adults feel connected with the world around them and feel that they still have a place in it. In addition, art-based practices in care and community promote older people’s well-being and the potential of increasing the quality of life of older people living in a long-term care facility and the community. This study has implications for evaluating arts ‘interventions’, not by using external goals, but conducting evaluation together with those involved, including the older adults and artists themselves.

## Figures and Tables

**Table 1:** Short description of the art projects in the study.

Art-Form	Description of Art Projects (Urban/Rural) (Scale Small, Intermediate or Large)	Average Amount of Older Participants
Dance, movement	1. Dance lessons + events in the community (urban, large)	• 15
	2. Dance labs to work towards a performance for older adults in long term care facilities (urban, intermediate)	• 10
	3. Weekly dance classes in long term care facilities (rural, small)	• 9
	4. Weekly dance classes with residents with Parkinson in a long term care facility (rural, small)	• 8
Music, singing	5. Community choirs (urban, small)	• 12
	6. Choir for people with and without dementia working towards a performance (rural, large)	• 30
	7. Choir with residents of an elderly care facility working towards a performance (urban, intermediate)	• 10
	8. Weekly band rehearsals (urban, intermediate)	• 8
	9. Series of improvisational music sessions for people with dementia and their carers in care facilities (rural, large)	• 15
Theatre, improvisation, performance	10. Weekly improvisational theater sessions in the community (rural, intermediate)	• 14
	11. Weekly theater rehearsals working towards a devised performance for older adults in care settings (rural, small)	• 12
	12. Weekly theatre labs for people living with dementia in a long term care facility (urban, intermediate)	• 7
Visual art	13. Walk-in intergenerational art studio in the community (urban, intermediate)	• 8
	14. Weekly intergenerational meetings focusing on drawing (urban, intermediate)	• 11
	15. Photography workshops with older people and secondary school students (urban, intermediate)	• 14
	16. Series of photography workshops in the community (urban, small)	• 10
Video	17. Weekly meetings to make a film in the community (rural, large)	• 15
Spoken word	18. Weekly spoken word workshops with people with dementia (urban, intermediate)	• 8

**Table 2:** Data collection of micro-narratives about the value perceived by older adults.

Number	Method	Participants	Data Format	Aim of Method	Period
Ia	Face-to-face semi-structured interviews	Artists (*n =* 20)	Transcripts	Building relations with leading artists and generating insights into the value of older adults from the perspective of artists	January–August, 2020
Ib	Interviews by telephone	Older adults (*n =* 8)	Transcripts	Gathering experiences of older people themselves, from those who were cognitively able to verbalize and reflect on their experience	
II	Semi-structured interviews by telephone	Older adults (*n =* 71)	Transcripts		
III	Participatory observations of the arts activities	Older adults	Field notes	Including stories of participants of art projects who were verbally or cognitively less able to tell researchers about their experiences. Getting a sense of the effect and experience of participants during the art activity itself, those aspects which might be harder to put into words or recall afterward.	
IV	Informal open interviews (3–15 min) during the participatory observations	Older participants, artists and (in)formal care professionals involved in these activities (*n =* 37)	Field notes	Gathering stories and experiences ‘in the moment’ of the arts activity	
V	Online workshops with arts-based techniques	Artists (*n =* 16)	Photography	To capture more subtle elements of participant experience harder to put into words, or cognitively reflect upon	September 2020–April 2021

**Table 3:** Phases of data analysis of this study.

Phase	Analysis	Means	Period
Inductive analysis to build a conceptual framework			
A	Thematic analysis of transcripts of face-to-face semi-structured interviews artists (*n =* 20) (method Ia)	MAXQDA (version 2018)	January–April, 2020
B	Thematic analysis of transcripts of formal semi-structured interviews (*n =* 50) with older adults (method Ib)	MAXQDA (version 2018)	March–May, 2020
Deductive analysis based on a conceptual framework for SenseMaker (Supplemental IV in Supplementary Materials)			
C	Analysis of micro-narratives (*n =* 470 of data of a diversity of methods (method I–IV)	SenseMaker (version 2020)	May–November 2020
Collaborative data analysis			
D	Collaborative analysis of the findings per project or art-form (10 sessions with 2–4 artists per session)	Digital workshop	November–December 2020
E	Validation session with different stakeholders (*n =* 56) around arts & health in the Netherlands	Digital workshop	January 2021

## Supplementary Materials

The following are available online at https://www.mdpi.com/article/10.3390/ijerph18158222/s1, Supplemental I: roles and responsibilities in the research team, Supplemental II: Interaction between the data collection and data analysis, Supplemental III: Conceptual Framework as a Basis of SenseMaker list, Supplemental IV: Topic guide for SenseMaker with older adults, Supplemental V: Insights from phase A-E of the study.
