# Supplementary material for: The Value of Active Arts Engagement on Health and Well-Being of Older Adults: A Nation-Wide Participatory Study
Source: Int J Environ Res Public Health. 2021 Aug 3;18(15):8222. doi: 10.3390/ijerph18158222 (PMC8345976; doi:10.3390/ijerph18158222)
Supplement: Supplementary file 1 [file ijerph-18-08222-s001.zip › ijerph-1266533-supplementary.pdf]

International Journal of
Environmental Research and Public Health

MDPI

Supplemental I

Topic guide semi-structured interviews with artists

Primary goal of the conversation: getting to know each other, understanding context and local setting of the initiative. Secondary goal conversation: First look into working mechanisms and implementation/continuation of the projects.

Introduction

- Introductions
- Explaining goal of the conversation
- Audio and recording of the conversation
- Informed consent
- Getting to know each other
- Explain about the set-up of the research, goals, methods, idea of learning together.

Context of the initiative

- Tell us about the project?
- Can you tell something about the start of the initiative, how did it start, how did it become what it is today? (First idea, who was involved in the start, conditions (place, people, materials, participants, resources))
- Who is (has been) involved with this initiative (draw mind map on paper)?
- How do people feel related to the initiative….(Like they 'own' the project? Like they are 'involved' in the project?)
- What feeling do you have yourself about the initiative?
- We read in… (website, project plan) about the intentions of the initiative, could you elaborate on that?
- Could you tell something about the participants in the initiative? (How do you find them, how are they involved? Background of participants (age, SES, gender, care needs, network, etc.))

Value of art projects

- During this research project we will be exploring values of arts in care. Can you describe one or more stories in which you experienced value for participating older adults?

Working mechanisms

- If you could draw a line which visualizes the start of the initiative until now, how would you draw it? How did things happen? What were the highlights, depths, challenging periods, etc.
- What are factors that are beneficial to the initiative? What caused growth or development of the initiative?
- What are hindering factors which take down, paralyze, irritate the initiative, etc.?
- How do you involve participants in the initiative?

Implementation and continuation

- At the moment, the initiative is (for now) depending on funds. How do you look at that?

- What is needed for implementation and continuity?

Rounding off

- What was the core of what was discussed today for you?
- Do you have any tips or ideas for us?
- Thank you

Supplemental II

Roles and responsibilities in the research team

Name role	Action that belong to the role
Junior partipicatory researchers	The junior researchers (L.K. and Y.L.) were involved from the moment of funding. They maintained contacts with artists daily, contacted participating older adults, asked them to share stories, conducted the participatory observations, did the formal analysis, helped in organizing workshops, co-facilitated co-research groups, organized team meetings monthly, and co-authored this article.
Senior participatory researchers	The senior researchers (B.G.) conducted the funding acquisition together with other seniors (C.D., J.L.), maintained contacts with artists on a more strategic basis, the conceptualization of the research protocol, conducted the formal analysis, facilitated workshops, participated in co-research groups, and team meetings monthly, and aligned the multi-disciplinary team, first author of this article.
Scholary artist	The artist (J.S.) was involved in the funding acquisition, participated in team meetings monthly, organized the online workshops with arts-based techniques, and co-authorized this article.
Senior co-researchers	The seniors (J.M. and Y.L.) were actively involved in gathering data and analyzing the findings. They helped refine the research questions and methods and collect data through interviews and participatory observations. They also joined in the data analysis, reporting, and joined team meetings monthly, and co-authored this article.
Supervisors	In this study, three supervisors were available. T.A. supervised the complete research, J.L. supervised one junior researcher (L.K.), and C.D. supervised the other junior (Y.L.). They joined in the team meetings monthly, had supervision meetings with their supervisees, and co-authored this article. T.A. monitored the methodological quality and integrity, spurred team conversations, created communicative space for reflections on the results, and stimulated the team to reach intellectual and conceptual depth
Critical friends	In this study, three critical friends were involved from the start (M.H. and T.T.). They joined team meetings monthly, T.T. also conducted some interviews and participated in the formal analysis, and both co-authored this article.

Artists co-researchers	The group of artists includes the same artists that led the 18 projects. They assisted with access to the participants, advise on methods that suit the local context, selection of older adults, invitation to art projects for participatory observations, collaborative analysis of the findings in online sessions with arts-based techniques.

Supplemental III

Interaction between the data collection and data analysis

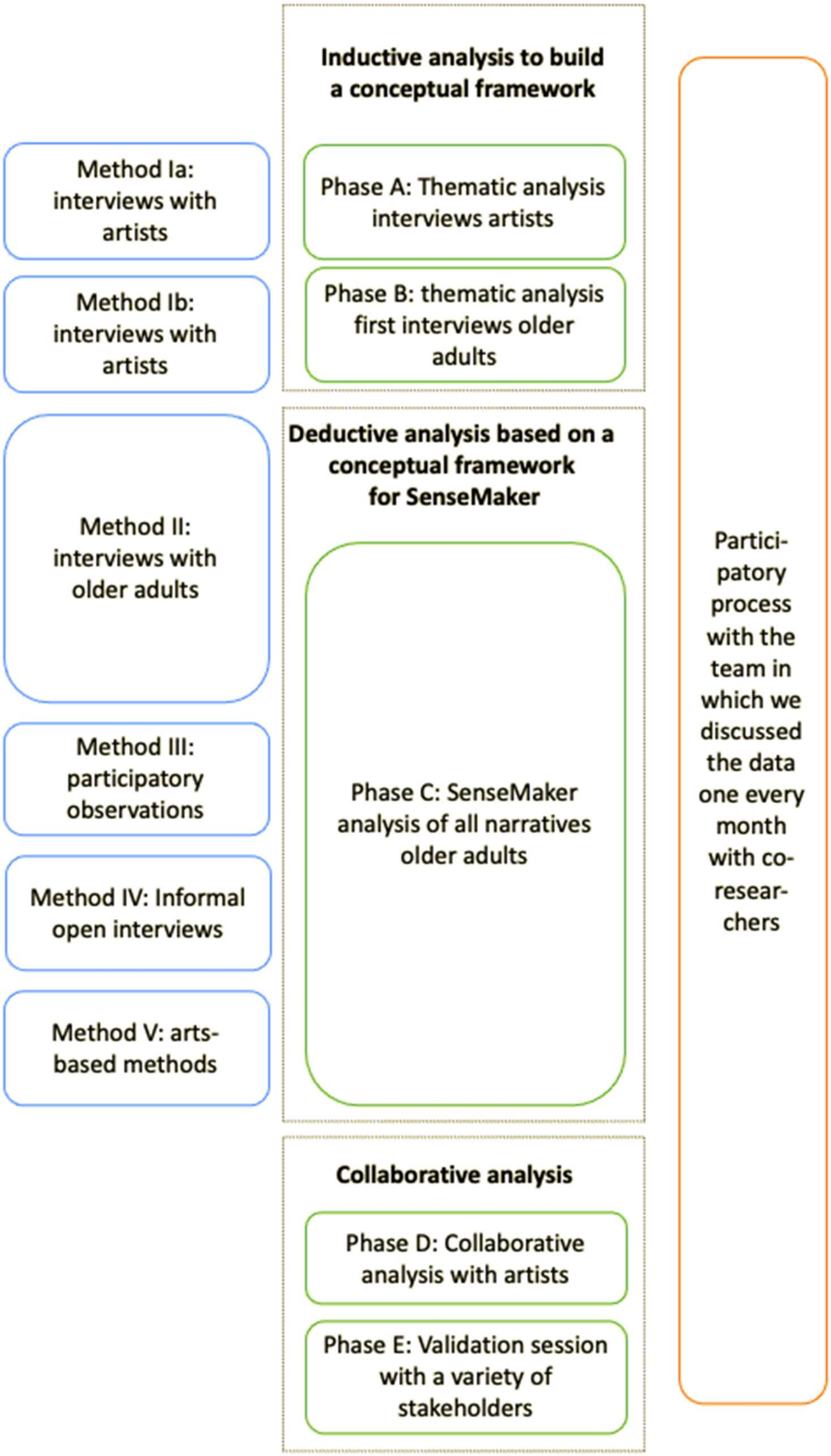

Supplemental IV

Conceptual Framework as a Basis of SenseMaker

Level	Concept	Subconcept	
Individual (being, becoming)	<u>Positive Health</u>	Meaningful living	Meaning to life
			Zest for life
			Being in a different world for a while
		Bodily functions	Feeling healthy
		Mental wellbeing	Being happy
			Accepting yourself
			Dealing with change
			Feeling better
			Less stress/fear
			Being accepted the way you are
		Quality of life	Breaking through regular routines
		Daily functioning	Dealing with time
	<u>Creative Health</u>	Feeling of freedom	
		Using creativity and fantasy	
		Being challenged	
Social (belonging)	<u>Positive Health</u>	Taking part	Social contacts, making new contacts
			Being taken seriously
			Doing fun things together
			Belonging
			Being amongst people
		Daily activities	Beter contact with carers and loved ones
	<u>Creative health</u>	Contact	Contact outside of regular social network
Societal (becoming)	<u>Inclusive society</u>	Imaging	Show who I am
			To mean something for someone else
			Change something about the image people have of older people

Supplemental V

Topic guide for SenseMaker with older adults

Share your experience

Share your experience! We are researching the role of arts in care. We would like to hear about a recent experience you have had with participating in an art project. We are gathering as many experiences as possible. In that way, we hope to learn more about the effect of active arts participation on the well-being of people.

Our request to you: Think back about a recent meeting or performance of a project in which you have participated. What did you experience that has stayed with you? Could you describe that experience and how you felt to us as if you were writing a diary entry? It could be a positive or a less positive experience. Which experience (positive or negative) comes up?

About your experience

We will be asking a couple of questions about the experience you have shared. Try to answer them all, but do not think about it for too long.

What would be a good title fort his experience?
<open answer>

What is your general feeling about this experience?

- Very negative
- Negative
- Neutral
- Positive
- Very positive

Which themes are central to you in this experience? (Max 3 answers!)

- Meaningful activity
- Feeling alive
- Keep on learning
- Bringing structure into the week or day
- Breaking through daily routines
- Meeting people
- Not applicable

For the questions below, click on the triangle and drag the ball to the correct balance between the three options, according to you. Or click 'not applicable'.

What is most important in your experience?

Relations with others

Physical aspects

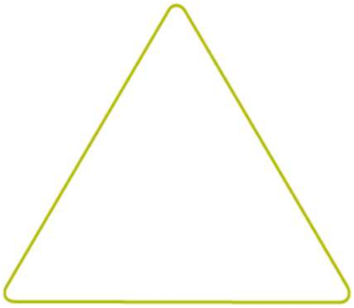

Feelings and emotions

o Not applicable

Which aspects of creativity do you see in your experience?

Feeling free

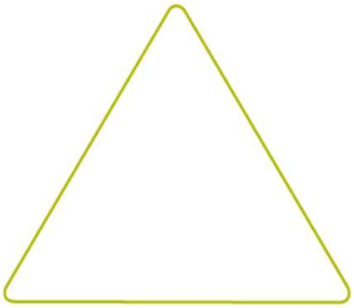

Using fantasy/imagination

Being challenged

o Not applicable

How does your experience relate to the body?

I experience my body differently

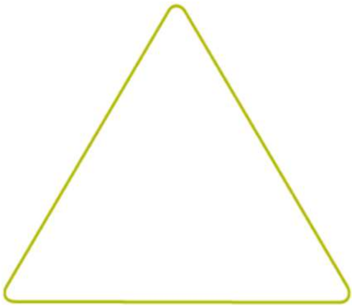

I have (more) confidence in my body

I feel physically more fit

o Not applicable

How does your experience relate to feelings? (Max 3 answers)

- It makes me happy and have fun
- Helps me deal with changes
- Helps me to accept myself
- Ensures that I experience less negative feelings
- Let's me be in another world for a moment

  Helps me to feel better
- Not applicable

About others

How does your experience relate to relations with others?

- Being able to show who I am
- Meaning something to somebody
- Changing something about the image people have of older people
- Not applicable

Which social aspects are important in your experience? (Multiple answers possible)

- Better contact with care providers,
- Informal carers or loved ones

- Contact outside of my usual social circle
- Make contact in a different way
- Contact outside of my usual social circle
- Being among people
- Be accepted as you are
- Belonging
- Doing fun things together
- Taken seriously
- Expand social network
- Not applicable

About you

How old are you?

- Younger than 50
- Between 50 and 60
- Between 60 and 70
- Between 70 and 80
- Between 80 and 90
- Older than 90

I am..

- Male
- Female
- Other

What is your living- and care situation?

- I live at home, without (professional)care
- I live at home with professional care
- I live at home, I receive informal care
- I live in an institution, independently
- I live in an institution and receive care
- Different, namely…

About the project

In which of the following arts projects are you involved?

- <names art projects>

What is your role in the project?

- Participant
- Initiator
- Artist
- Informal carer
- Professional carer
- Volunteer
- Different, namely…

Finally

Who filled out this questionnaire?

- I, myself
- A researcher
- Initiator, artist or volunteer
- Someone else, namely…

Are we allowed to (anonymously) share your story with others?

- Yes, my story can be anonymously shared with those interested.
- No, my story is only for directly involved researchers.

Supplemental VI

Insights from phase A-E of the study

Phase	Action	Results	Period
First analysis of data			
A	Thematic analysis of transcripts of face-to-face semi-structured interviews artists (N=20) (method I)	The themes on three levels: individual, social and societal level.<br>• **Individual**: 1) Growth process and development, 2) Opening to each person's feelings, 3) Physical and mental stimulation.<br>• **Social**: 1) Mutual affection, 2) Contact, 3) All other kind of connections.<br>• **Societal**: Image of older people	January – April, 2020
B	Thematic analysis of transcripts of formal semi-structured interviews (N=50) with older adults (method II)	The themes on three levels: individual, social and societal level.<br>• **Individual:** 1) Meaningfulness (meaningful life, zest for life, being in another world for a while), 2) Body functions (feeling healthy), 3) Mental well-being (being happy, accepting yourself, dealing with changes, feeling better, less stress/anxiety, being accepted as you are), 4) Quality of life (breaking the daily routine), 5) Daily functioning (dealing with time), 6) Feeling free, 7) Using creativity and imagination, 8) Being challenged.<br>• **Social**: 1) Participation (social contacts, being taken seriously, doing fun things together, belonging, being among people, 2) Daily activities (better contact with care providers), 3) Contact (contact outside one's own familiar circle)<br>• **Societal**: Image of older people (show who I am, mean something to others, change the image that people have of older people).	March - May, 2020
Making a conceptual framework for SenseMaker using analysis phase A and B, and academic literature (Supplemental III)			
C	Analysis of micro-narratives (N=470 of data of a diversity of methods (method I-IV)	The focus of the narratives focused on feelings, emotions and relations. The narratives focused on the individual and social level. Not many societal value was found in the narratives.<br><br>Key themes were:	May – November 2020

		• **Individual**: 1) Growth process and development, 2) Positive Feelings<br>• **Social**: Different way of contact	
Collaborative data analysis			
D	Collaborative analysis of the findings per project or art-form (10 sessions with artists)	Overall, artists underscore the findings of phase C. The artists alle argue that the value of the projects also relates to the art-form, for example, dancing also focusses on coordination or fitness.	November – December 2020
E	Validation session with different stakeholders (N=56) around arts & health in the Netherlands	Overall, artists underscore the findings of phase D. In the discussion the focus is on the small amount of stories focusing on the bodily experiences and affect.	January 2021
